# Experiences of people with long COVID: Symptoms, support strategies and the Long COVID Optimal Health Programme (LC‐OHP)

**DOI:** 10.1111/hex.13879

**Published:** 2023-09-26

**Authors:** Hiyam Al‐Jabr, David R. Thompson, David J. Castle, Chantal F. Ski

**Affiliations:** ^1^ Primary Community and Social Care Faculty of Medicine and Health Sciences University of Keele Keele UK; ^2^ School of Nursing and Midwifery Queen's University Belfast Belfast UK; ^3^ Department of Psychiatry University of Tasmania Hobart Tasmania Australia; ^4^ Centre for Mental Health Service Innovation Hobart Tasmania Australia

**Keywords:** COVID‐19, long COVID, mental health, Optimal Health Programme, patient experience, qualitative research

## Abstract

**Introduction:**

Long COVID (LC) is a multisystem illness, with fluctuating symptoms that affect the daily activities of patients. There are still no standardised diagnostic criteria or treatment approaches for managing LC. The LC‐Optimal Health Programme (LC‐OHP) was designed to support the mental wellbeing and physical health of people with LC. Gaining an in‐depth understanding of patients' experiences and support strategies is imperative to identifying appropriate supports to guide them through their recovery. This study aimed to elicit the experiences and perceptions of adults with LC regarding symptoms, support strategies and the LC‐OHP.

**Methods:**

As part of a wider randomised controlled trial of the LC‐OHP, participants in the intervention group had their sessions audio‐recorded. Transcripts were thematically analysed to identify common emergent themes.

**Findings:**

The LC‐OHP was delivered to 26 participants. Data were collected between January 2022 and February 2023. Four main themes emerged: ‘Symptoms and impact of LC’; ‘Other sources of support and perceived challenges’; ‘Strategies to support LC’ and ‘Perceptions of the LC‐OHP’.

**Conclusion:**

LC experiences were mostly described as fluctuating and burdensome that significantly impacted daily activities, and physical and mental health. The LC‐OHP was perceived as beneficial. Access and experiences of other sources of support were varied. Increasing LC awareness amongst health practitioners and the wider community has the potential to improve the experiences of those affected by LC.

**Patient or Public Contribution:**

The LC‐OHP was derived from the OHP. It was adapted to people with LC following consultation with practitioners at an LC clinic. Additionally, the mode and timing of delivering the programme to this population were taken into account for its delivery at the convenience of participating patients. While considering that fatigue and brain fog are amongst the most reported complaints of people with LC, public members with LC were not involved directly in this study; however, feedback obtained from practitioners working with this population was implemented in amending the programme and its delivery. Additionally, feedback from patients with other chronic health conditions who used the OHP in previous studies has been implemented to make the programme more user‐friendly. Moreover, feedback obtained from participants receiving this programme in this study was implanted immediately and shared with other participants. Finally, this study was overviewed by a data management committee that included two public members with LC, who contributed and provided guidance to support this study.

## 1 INTRODUCTION

As of June 2023, more than 767 million cases and 6 million deaths resulting from the COVID‐19 global pandemic have been confirmed.^1^ In the months following the start of the pandemic, it became apparent that many symptoms of this new infection were persistent for weeks, even months, beyond the acute infection; this phenomenon has been referred to as long COVID (LC).^2^

LC is a complex, multifaceted illness with numerous, cyclical and variable symptoms manifesting over a relapsing and remitting course.^3^, ^4^, ^5^, ^6^ There remain many unknowns surrounding LC, for example, whom it might affect and how long it will last.^7^ However, several risk factors for LC have been identified including female sex, old age, pre‐existing comorbidities and obesity.^8^, ^9^, ^10^, ^11^, ^12^, ^13^, ^14^, ^15^, ^16^ With limited evidence on effective treatment interventions and wide‐ranging and fluctuating symptomology, there is no clear standardised approach to the diagnosis and management of LC.^7^, ^17^, ^18^, ^19^, ^20^, ^21^ The only consensus is that a comprehensive management approach should be applied.^5^

To date, there is no unified definition for LC.^22^ For example, in the United Kingdom, the National Institute for Health and Care Excellence (NICE) defines LC as encompassing both ongoing symptomatic COVID‐19 (from 4 to 12 weeks) and post‐COVID‐19 syndrome (12 weeks or more), in the absence of an alternative diagnosis.^5^, ^23^ In the United States, however, LC refers to the post‐COVID‐19 condition taking place usually 3 months from the onset of the acute COVID‐19, and with symptoms lasting 2 months or more.^24^ In practice, LC is used as an umbrella term that covers a wide range of post‐COVID symptoms.

LC imposes many limitations, including physical and mental health, financial burden and social restrictions.^25^ The effects of LC have been described as *crippling and life‐changing*.^26^ Most commonly reported symptoms include fatigue^9^, ^14^, ^27^, ^28^ and neuropsychiatric symptoms (e.g., anosmia, brain fog and neuropathy).^7^, ^29^ In the absence of clear diagnostic and treatment pathways, along with social stigmas, patients' mental health (e.g., stress and anxiety) is often severely impacted.^30^ As a relatively new condition, the research on the lived experiences with LC and its associated challenges is still restricted yet evolving. A recent systematic review identified a number of studies, with people describing LC as a heterogeneous condition, with a variety of physical and emotional consequences.^31^ To best support those affected by LC, a comprehensive understanding of people's experiences with LC is essential.^32^, ^33^

This study is part of a wider randomised clinical trial that assessed the feasibility of the Long COVID Optimal Health Programme (LC‐OHP), a psychosocial educational support programme for mental and physical wellbeing customised for people with LC.^34^, ^35^ Participants in this study were those randomised to the intervention group. The LC‐OHP was adapted from the OHP originally designed to support and benefit the physical and mental wellbeing of patients with long‐term medical conditions.^36^ It employs a holistic, person‐centred approach to support patient self‐management^36^ delivered in weekly sessions that address specific aspects of health and wellbeing most impacted by LC. The programme focuses on health as defined by individual patients. It supports patients' self‐efficacy and self‐management skills, and works on shifting their focus from being ‘dependent on services’ to being ‘supported by services’,^37^ consequently, contributing to reducing pressure and financial burdens on healthcare systems. The key elements of the programme are summarised in Table 1. This study reports on participants' experiences during the LC‐OHP sessions.

**Table 1:** Long COVID OHP programme sessions.

Session	Title	Content
**1**	Optimal health	What is optimal health?
		Optimal health wheel
**2**	I‐Can‐Do‐Model	Strengths and Vulnerabilities
		Stressors and Strategies
		Health plans 1 and 2
**3**	Factors of wellbeing	Medication and metabolic monitoring
		Collaborative partners and strategies
		Health plan 3
**4**	Visioning and goal‐setting	Defining change
		Orientation and preparation
		Creative problem‐solving and goal‐setting
		Reflection and celebration
**5**	Building health plans	Health plans 1, 2 and 3
		*My Health Journal*
Booster	Reflecting on the learning in the transformational journey to sustain wellbeing	Reflecting on the learning in the transformational journey to sustain wellbeing

Abbreviation: OHP, Optimal Health Programme.

### 1.1 Aim

To report the experiences and perceptions of adults with LC of their symptoms, support strategies and the LC‐OHP.

### 1.2 Governance and ethical approvals

Ethical approval for the study was granted by the university and Health Research Authority ethical committees and was overseen by a data management committee that included public members. The study protocol and outcomes from the randomised controlled trial (RCT) are reported separately.^34^, ^38^, ^39^

## 2 MATERIALS AND METHODS

This study is part of an RCT that was conducted at a local university in the United Kingdom between December 2021 and May 2023. The study received ethical approval from the University and from the Health Research Authority ethics committees. Potentially eligible participants were recruited via social media and through referrals from LC clinics. Eligible participants for the trial were those aged 18 years and above, have LC and can communicate in the English language. Participants were consented (signed written consents after reading the study information leaflet) and randomised to either the control group (usual care) or intervention group (LC‐OHP). Additionally, for this study, participants were those who attended the minimal number of LC‐OHP programme sessions (five sessions) between January 2022 and February 2023. All sessions were audio‐recorded for note‐taking and to facilitate discussions. Participants were informed in the information leaflet and consent form about the recordings that will be used for data analysis. Participants were reminded and verbally consented at the start of each session to record the session.

No topic guide was used as participants were not interviewed. Rather, discussions at each session were guided by the key elements of the programme described in Table 1. This study reports on the dialogues and notes taken during the sessions. Sessions were delivered by the same researcher (H. A.‐J.) and were delivered remotely (virtually or via telephone) at a convenient time.

The researcher listened to the recordings and took notes of participants' experiences at each session. Sessions' notes were anonymised, coded and thematically analysed (using inductive thematic analysis).^40^ Notes were revisited, and accuracy was verified by comparison of notes and recordings. Relationships between codes were established and similar codes were grouped together to develop subthemes and final themes; these were checked by other members of the research team to identify common emergent themes and to ensure appropriate and consistent coding processes. Any disagreements were resolved by consensus, and by referring to the notes and original recordings. Final themes were presented to the research team and were supported by anonymised quotes from the different participants. The coding of data was conducted using ATLAS‐TI software (Version 23.1.1.0) and was guided by reaching data saturation, that is, when no new themes were emerging from the data.

To ensure rigour with study activities, the researcher who delivered the sessions received prior training on delivering the programme during a two‐day workshop provided by the programme developer (D. J. C.). Regular supervisions and debriefings by other members of the research team were also provided, and a fidelity checklist was completed after each session by the researcher and by another member of the research team (who listened to the recordings), to ensure all core components of each session have been covered. The researcher has a professional background in pharmacy and research experience in interacting with patients with different medical conditions.

### 2.1 Findings

The wider trial recruited 60 participants, of which, 28 were randomised to the intervention group. A total of 163 sessions were delivered to 26 patients. The two other participants withdrew from the study due to health issues, they were not included in this part of the study. Out of the 26 participants, 19 completed a minimum of 6 sessions (6–10 sessions in total). Sessions took between 30 and 90 min and were delivered 1:1.

Most participants had LC for several months (mean 11 months; range 4–28). The study cohort included 2 males and 24 females, aged between 21 and 69 years (mean = 46.8 years; SD = 13). The majority were of white British ethnicity (*n* = 22, 84.6%), and held an undergraduate degree. The most common method of participant recruitment was through social media, for example, Facebook, Twitter and Instagram (see Table 2).

**Table 2:** Participant characteristics and recruitment methods.

Characteristic	Total no. (%)
Age, years	
18–29	3 (11.5%)
30–39	5 (19.2%)
40–49	6 (23.1%)
50–59	7 (27%)
≥60	5 (19.2%)
Gender	
Female	24 (92.3)
Male	2 (7.7%)
Ethnicity	
White British	22 (84.6%)
Other White backgrounds	4 (15.4%)
Education	
Secondary education	1 (3.8%)
Postsecondary education	5 (19.2%)
Undergraduate degree	9 (34.6%)
Postgraduate degree	7 (27%)
Vocational qualification	4 (15.4%)
Recruitment method	
Referral from an LC clinic	5 (19.2%)
Social media	12 (46.2%)
Research studies website	6 (23.1%)
Other LC support groups	3 (11.5%)

Abbreviation: LC, long COVID.

**Figure 1:**
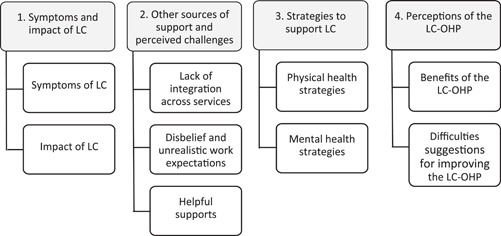
Summary of main themes and subthemes. LC, long COVID; OHP, Optimal Health Programme.

Thematic analysis identified four key themes and associated subthemes. A summary is provided in Figure 1.

### 2.2 Theme 1: Symptoms and impact of LC

Participants described LC as a confusing illness, surrounded by uncertainty in clinical pathways, randomness in presenting complaints and associated with a wide range of complex and fluctuating symptoms encompassing the nervous, cognitive, cardiovascular and muscular systems. Participants also reported several impacts of LC that influenced various aspects of their lives.

#### 2.2.1 Symptoms of LC

Participants expressed varying degrees of brain fog and mental fatigue, including feeling overwhelmed, having concentration and memory difficulties, feeling sensitive to noise and unable to comprehend people talking quickly, and difficulties with reading, finding words and processing information.

Debilitating levels of fatigue were reported by most participants, impacting their ability to carry out daily activities. Fluctuations in fatigue made it difficult to plan day‐to‐day activities. Some participants described a notable muscle deterioration causing them to be wheelchaired or homebound, thus, limiting their activities, compared to pre‐LC.

…when I go out now, I have a wheelchair, I can't push my eight‐year‐old daughter on the swings, can't walk my dog, and obviously, can't go to work until the issues with my heart rate has been sorted… [Participant‐G]

Other symptoms mentioned by participants included skin rashes and allergies, frequent headaches and/or migraines, changes to their voice (developing an ‘LC voice’), shortness of breath when laying down or talking, difficulty breathing, tinnitus, ear pain and vertigo.

#### 2.2.2 Impact of LC

Many participants described how LC impacted their sense of identity, leaving them with feelings of uncertainty, for their sense of self and future, noting differences to pre‐LC. Participants expressed changes in social roles as they became receivers of care, compared to previous roles of caregiver and/or providing support to family members.

Before covid, I've sort always been the one to look after everyone and the calming, reasonable, logical person, sort everything out, and I've had issues with health stuff in the past, and I always found a way to find ways around things … however now, there are so many things it has impacted‐they all make each other worse. [Participant‐Z]

The impact of LC on mental health was felt by most. Participants spoke of feeling stressed, anxious, sad, and fearful of an unknown future; *is this ever going to get better?* Some described a notable decrease in their stress threshold, while some reported effects on diet, for example, new or exacerbated food allergies and intolerances, a decreased ‘hunger radar’ and appetite, reduced food intake and changes to taste and smell. Other participants reported heart symptoms, most notably postural hypotension and developing postural orthostatic tachycardia syndrome (POTS). Sleep disturbances were also mentioned, including insomnia, variations in sleep duration, nightmares and vivid dreams, reduced sleep quality, frequent naps and morning headaches.

Many participants also mentioned the impact of LC on their work and how they had to take prolonged sick leave, with most feeling stressed and worried, especially for being pressured to get back to work. A few participants were on a phased return‐to‐work plan.

### 2.3 Theme 2: Other sources of support and perceived challenges

Participants reported seeking a range of supports for additional care before receiving the LC‐OHP. Supports included family and friends, healthcare professionals (HCPs), social media, work, LC support groups and LC research studies. Participants' perceptions of these supports were varied, reflecting both positive and negative experiences.

#### 2.3.1 Lack of integration across services

For the majority, participants described not receiving the support they expected to receive from their HCPs and several challenges with health services. Accessing the healthcare system was described by many as a lottery with LC supports described as ‘fragmented’, ‘siloed’ and ‘long waiting periods’ for access. As participants had to be referred to an LC clinic by their general practitioners (GPs), with most waiting several months for an appointment, many turned to the private health sector, finances permitting, to circumvent lengthy waiting periods.

Participants spoke of GP appointments with different GPs, highlighting the physical and mental strain of having to repeat their story several times over. This culminated in feelings of ‘loss of continuity of care’ described as increasing their levels of exhaustion, because their stories were often long and complex.

…I've done long‐term management of conditions for years, even the GP system is broken, because you cannot have contact with the same GP… I really would like to … see the same GP face‐to‐face … my doctor surgery got probably 30+ doctors …. a lot of long covid patients I met through the groups said the same thing, I'm really frustrated having to phone first thing in the morning, wait around all day for someone to phone you back, to have a 7 min phone call, and have to explain it all again, and some of them I've got like two year journey with long covid or longer … it's just not making long‐term medical conditions management … it's not easy if you don't have the same person [GP], it makes it 10 times harder. [Participant‐R]

Other participants mentioned a lack of communication between HCPs. A repercussion was that participants had to repeat their LC story, a situation that was worsened for those with multiple medical conditions. Some felt due to the lack of HCP communication, advice given by practitioners could be detrimental and/or conflict with treatments received for other conditions.

…because you are not speaking to the same person … like urology is treating one part of me, it's just one part of the jigsaw, I wish somebody would look at me as a whole, because like I said, for my POTS and heart increase the salt but because of my urology issues and it's like no one's … so I'm getting conflicting treatment and then I have to sit down and go ‘but I can't do this’, and then I have to double check it with them and then … but I'm not medical I'm not a medical professional, I know my body, but I'm not medically trained … so I'm having to read about conditions but literally … it's like written in another language … it's written in medical terminology. [Participant‐R]

Participants also acknowledged challenges encountered by the healthcare system started long before the pandemic, acknowledging that the NHS was already overwhelmed and underfunded. Many also described HCPs as not having the necessary tools to support LC, busy schedules and minimal knowledge or an incomplete understanding of LC. Some participants highlighted the importance of using a holistic approach to manage LC. Suggestions included a fast‐tracking system for managing staff with LC so that they would be able to get back to work to support others.

I also find it ridiculous that they…they're short staffed enough within the NHS, and they have no fast‐track service or priority for staff or separate service for the staff to access that would allow them to be back at work to then treat more people to clear the backlog, but that's the NHS … it's completely short‐sighted … I think they're not helping themselves by not looking after their staff. [Participant‐K]

#### 2.3.2 Disbelief and unrealistic work expectations

Participants frequently expressed distress when they encountered situations where their symptoms were not acknowledged, validated or believed by their HCPs. Some reported not receiving adequate advice on what to do with their LC and not receiving holistic care. Some felt that GPs only focused on prescribing medications and had a lack of understanding of LC. They expressed an overall sense of lack of, or insufficient LC support from HCPs and the healthcare system. Others felt a sense of abandonment, with no option but to sort things out by themselves by seeking other forms of support.

…the model of ill health that they [GPs] work with doesn't encompass a holistic approach to understanding your patient …. none of the things we've been talking about have been raised with doctor [name], to be honest I wouldn't expect them to, at all, because I just don't think they have time or the skills to help me… [Participant‐M]

…occupational health has been understanding yet not very useful, the long covid clinic was a complete waste of time, and my GP … basically said ‘well I don't know what else to suggest’. So, I kind of feel completely abandoned by the health team and left to kind of sort it out myself. [Participant‐K]

Many participants criticised the healthcare system for the way the pandemic was handled. For example, some participants were NHS staff members, and they felt pressured to get back to work, especially as this was in direct contradiction with LC recommendations for promoting good health.

…it is a bit bizarre to be working for an organisation that is trying to promote health, and yet they are not allowing me to do what the professional advice would be … I'm doing what I wouldn't advice patients to do… I haven't got the time anymore cause I only got till the end of [month] to establish what I can and cannot do… [Participant‐F]

The impact of LC on work was also discussed by participants. Many reported experiencing periods of sick leave that would last for several months, some were on a phased return to work, while others had left work to focus on their recovery. Supports provided by workplaces included handing over tasks that could be covered by other colleagues, allowing for remote working and offering alternative roles to engage in while recovering. Although some described a positive working environment and support, the general sense was a lack of support and pressure to get back to work. Many felt this impeded their recovery, through stress‐related concerns regarding their work, future and finances.

Participants spoke of stereotyping and disbelief by family, friends and GPs, highlighting a lack of knowledge, understanding and even a denial of the existence of LC. One participant reported they also had to deal with stereotyping of being young, that is, being fit and healthy.

…yesterday my sister came with me to pick my meds, we had a walk up a flight of stairs, and I was getting sort of out of breath and dizzy on the stairs so I did my usual, just stopped for a minute and I didn't realise there happens to be a women behind me, and the woman behind me huffed and got really annoyed, and sort of went pass me and carried on huffing and my sister bless her she got so frustrated and annoyed at this woman and I was like she doesn't know … she doesn't know what's going on, she just thinks I'm being nuisance that I stood in her way. [Participant‐Z]

Further to this, some participants felt that their friends did not want to know or become involved in their situation, possibly because of discomfort listening to their travails, and because they could no longer engage in the activities they were accustomed to. Others reported accusations of ‘making it up’ as a pretext for claiming time off work.

#### 2.3.3 Helpful supports

On the other hand, there were some participants who described positive, therapeutic relationships with their HCPs. They described being listened to, believed and validated for what they were going through. Varied practitioners were recognised, including GPs and practitioners working in cardiology, physiotherapy, neurology, ear, nose and throat, occupational health, LC assessment services, nutrition, speech and language therapy, respiratory, endocrinology, rehabilitation, myalgic encephalomyelitis/chronic fatigue syndrome and psychology. Some expressed having easy access to their GP practice and frequent appointments as a helpful source of LC support.

Social media was another source of support participants highlighted. Most agreed that online LC‐peer groups provided a useful source of information and support, especially with the isolating nature of the illness. Validation of symptoms was achieved through the sharing of LC experiences and exchanges of knowledge and information.

Support from family and friends was mentioned, that is, social, emotional and financial support, adjustments to living environments, as well as additional medical support and advice from those with various healthcare qualifications (e.g., nursing, physiotherapy). However, many participants expressed not having such forms of support, with some adding that their families lived far away, thus adding to their sense of isolation. Also expressed was that their symptoms were not acknowledged or validated by family members and/or friends, making their situation more difficult.

having a network is sometimes easier said than done, especially when you live on your own and your family lives 10 miles away from you, I mean, you know, for some people that's the reality. [Participant‐N]

Other forms of support described by participants included: LC support groups in the workplace; LC research studies; social media and podcasts; applications designed to support LC; LC relaxation exercises; medications recommended by others affected by LC (e.g., antihistamines); herbal therapies, acupuncture, cranial osteopathy, hypnotherapy, cognitive behavioural therapy and one mentioned using a mobility scooter to aid with transportation.

### 2.4 Theme 3: Strategies to support LC

Participants shared a wide range of strategies to help them manage their LC symptoms, especially fatigue, mental health and physical symptoms.

#### 2.4.1 Physical health strategies

Regarding their physical health, participants talked about going through cycles of fluctuating energy levels and activities. Many described increasing physical activity on their ‘good days’ would inevitably lead to post‐exertional malaise that could extend from days to weeks. Strategies to help deal with physical symptoms included: using a mobility scooter; breaking down tasks into smaller ones; pacing and planning, being strict to avoid overexertion (e.g., one activity a day, breaks between activities, relaxation exercises); participating in exercise groups specific to LC and monitoring and tracking activities (e.g., using a Fitbit).

For me it's a case of bearing that in mind constantly and perhaps you could have a posted in your room saying ‘don't get carried away … be patient’, …. ‘There is obviously, one of these trials for LC that I've seen they use texting to tell you that your heart rate reaches a certain threshold and a text message to tell you that you need to slow down…’…. ‘Actually using breathing techniques I think is quite effective, I found that if I'm able to take longer inhales and exhales …. actually that helped … it brought down my heart rate while I was walking’. [Participant‐D]

Other strategies reported include monitoring fatigue and energy levels, taking frequent rest periods, making suitable adjustments to home and working environments to reduce energy expenditure, taking painkillers before doing physical activity and walking with someone. Some participants also reported taking vitamin B12 and folic acid to replenish deficiencies and thus improve their fatigue levels.

#### 2.4.2 Mental health strategies

Strategies related to neurological symptoms included speaking to other people, using reminders, writing notes, deep breathing, planning, listening to the radio instead of reading, avoiding noisy areas, muting TV to reduce noise, completing word puzzles and placing notes in a glass jar or on a board in acknowledgement of any positive achievements.

I tend to listen to calming music and listen to audio books, I also do … on Netflix they have the headspace guided meditations and stuff, I do those, because like I say pre‐covid I would read I would draw things like that, but the difficulty is now with my fatigue and my brain fog, concentrating on those things can be hard, and also like drawing and painting… I sort have to prioritise that for work now rather than leisure as well because I get shaky hands I get pain, so I had to prioritise, and I had to readapt on how to relax and calm myself. [Participant‐ H]

Several participants reported difficulties with planning ahead due to symptoms, such as brain fog and fatigue. However, a wide range of strategies was reported as helpful, for example, planning with other family members, cooking in bulk, planning as much in advance as possible, assessing fatigue levels to decide whether to take a resting day, prioritising three positive things to do every day and planning daily and weekly rest periods to preserve energy levels.

Participants also talked about strategies to help them deal with LC acceptance and stress management. For example, being realistic and lowering expectations, using distraction techniques to divert thinking away from their illness, speaking to others, accepting their situation—what they can and cannot do, being optimistic about progress, increasing their understanding of what absorbs most energy and recognising incremental progressions.

### 2.5 Theme 4: Perceptions of the LC‐OHP

The LC‐OHP was generally well received by participants. Several benefits and positive experiences were reported in mitigating encountered symptoms. Few participants also reported difficulties with the programme.

#### 2.5.1 Benefits of the LC‐OHP

Beneficial aspects of the programme included activities that helped them learn more and gain a better understanding of LC and how to manage their symptoms. They described how the LC‐OHP acted as a tool for enabling a shift of perspective, for example, approaching LC holistically, rather than focusing on certain symptoms. It also helped them to improve their coping mechanisms by building a positive mindset, for example, focusing on small, positive steps towards recovery, rather than comparing how they used to be pre‐LC. The programme also made participants appreciate the support from family, friends and their environment, through various tools that helped to visualise and identify their supportive network.

Participants highlighted the importance of how the LC‐OHP provided an opportunity to share their experiences with someone else, to be listened to, not to be judged and to gain validation on their LC journey and the activities they engaged with to support their self‐management. Some participants found that only through the LC‐OHP they had learnt about activities that aided their control of and support for LC symptoms.

I think it was a learning curve to do the pacing but it was also a learning curve to…although I understood before how stress can affect the body, to have my body affected so badly in so many physical ways by things that I haven't even acknowledged were stressful was a really big learning curve and I think this was part of what you address in this programme and it's really helpful to understand that…. [Participant‐C]

The programme helped many participants change their behaviour by adopting healthier ones (e.g., avoid pushing self) to maintain their progress. These strategies enabled many to boost their energy levels, identify what they could and could not do, recognise limitations and adjust activity levels accordingly. It also assisted with altering their mindset to reach a level of acceptance, therefore, helping them to regain some control over their life.

I would be a great advocate for this programme because it's…it sort of captures all of me, so… and I felt really supported throughout the programme, it made me make an element of commitment because I knew there were things I needed to do in order to have that conversation, you know preparation or whatever. I found it very therapeutic, and it taught me things, and also taught me acceptance about you know the condition that I've got and it'll take time and I'll probably get better eventually, but I find the programme incredibly supportive in way which it was structured, it took you in a journey, which I found really helpful. [Participant‐P]

Participants described how the holistic nature of the programme helped, they could use it not only for managing their LC but also for planning and managing other aspects of their life, such as their working environment. Rather than learning through trial and error, the programme pinpoints important strategies for better management of their condition. Participants also made suggestions of referring GPs to using the LC‐OHP along with regular check‐ups.

#### 2.5.2 Difficulties and suggestions for improving the LC‐OHP

Although participants found the programme activities helpful, some expressed difficulty with completing the weekly tasks for the programme, especially because of their unpredictable symptoms and energy levels. Certain elements of the programme were also not easy to understand, thus needing further clarification/detail.

Additionally, many participants agreed that it would have been more helpful to receive the programme at an earlier stage of their illness, as these participants expressed knowledge of some programme elements because they already were proactively searching for other support from various resources.

I think it … even though…. I had kind come to all the things in the booklet for myself, that's because I'm interested in psychology… for me because I'm am lucky and I am positive, I have a nice support group, if you didn't have that your programme would be so … more valuable, for me it was validating and I really appreciated it … i think for the programme as a whole the earlier that people could get it the more benefit they would get from it. [Participant‐G]

## 3 DISCUSSION

This study is the first to report on the experiences and perceptions of managing symptoms of LC, along with their experiences of a holistic intervention, the LC‐OHP. These findings identify the impacts of LC on daily life activities, various sources of support, struggles encountered with HCPs and the community and experiences of the LC‐OHP.

Overall, the findings of this study are consistent with those of other studies with regard to the lived experiences of people with LC^17^, ^41^, ^42^, ^43^, ^44^ and with symptoms listed in the Scottish Intercollegiate Guidelines Network and the NICE 2021.^32^, ^45^, ^46^, ^47^, ^48^ Although participants' experiences with LC in this study were unique and affected by various factors, themes identified reflected consistencies across their experiences. Recovery from LC was reported as a long journey with fluctuating symptoms affecting physical health and mental wellbeing. The wide range of LC symptoms necessitates using a comprehensive, person‐centred approach in supporting patients.

Regarding healthcare services, many struggles were reported, including difficulty accessing care, being disbelieved, and not receiving the care they expected to receive and care provided was generally described as being fragmented—not unsimilar to other studies.^7^, ^17^, ^49^ Although many participants acknowledged pre‐existing challenges facing healthcare systems, the uncertainty surrounding LC, and that HCPs often do not have the tools to manage this condition, participants expected them to provide comprehensive care and listen to and validate their symptoms. Providing person‐centred, holistic care to people with LC was recommended from the start of the pandemic.^48^ However, the findings of this study indicate that there are still challenges with providing such care. Working closely with HCPs to identify barriers in managing LC shows potential for identifying best‐suited LC support strategies. Providing HCPs with further training and educational sessions on LC may assist with increasing awareness to better equip them to support their patients. The LC‐OHP shows much potential for use to support such training to guide practitioners towards a comprehensive, consistent, yet personalised approach to support people with LC.

These findings demonstrate the importance of improving communication skills, as many participants reflected poor interactions with HCPs, where they felt not listened to or validated. Effective use of communication skills is recommended by the General Medical Council's standards of professional practice,^50^ with HCPs being encouraged to engage reflexively with their patients.^51^, ^52^

Participants spoke of the challenge of having to repeat their LC story at each GP appointment, and with fatigue and brain fog, this became increasingly difficult. As similar burdens have been reported in other studies,^17^ identifying measures that could be put into practice to schedule appointments with the same GP should be prioritised. There was also a general sense that participants' symptoms were not acknowledged or validated by family and/or friends.^32^ This lack of validation was seen as an additional burden, further negatively impacting their mental health. Previous studies reported that self‐adjustment and support from family and social networks are key to helping people with infectious diseases.^53^, ^54^, ^55^ Thus, increasing awareness of LC for practitioners, family and friends is required to improve understanding and provide better support.

Similar to other studies,^32^, ^49^ seeking LC support was described as a necessity. Our findings highlighted the importance of peer support and social media regarding validation and acknowledgement of their LC journey. Considering the isolating nature of LC, this type of support was seen as providing participants with opportunities to share and listen to the experiences of others, and importantly provide a form of validation. Furthermore, sharing lived experiences can inform others including practitioners, allowing them to gain a better understanding of LC.^32^

Participants reflected on being proactive during extended periods between care appointments, by trying alternative treatment strategies to speed their recovery. A wide range of strategies to manage their LC symptoms were reported, that is, those related to fatigue, mental and physical health. Some strategies were described as having multiple effects on various symptoms, that is, planning regular rest times. Participants also reported sharing the strategies and knowledge they gained through various avenues, which again indicates the need to increase awareness of LC, symptoms and possible treatments.

In regard to returning to work, recommendations to support those with LC include ensuring this is in accordance with their recovery.^56^, ^57^ In our study, some participants noted receiving various forms of support, while others felt pressured to get back to work. The latter expressed that the lack of support only made their situation more stressful as they had minimal recovery time. In this regard, employers should be supporting staff to return to work on their terms, that is, when they feel able.

Participants also reflected on their experiences with the LC‐OHP describing components of the LC‐OHP that helped them to better manage their LC symptoms. For example, providing participants with various tools to monitor their daily activities and energy levels allows them to better plan accordingly. The LC‐OHP also encouraged them to adopt healthier behaviours by gaining a new perspective on their illness, that is, focusing on their achievements since acquiring it. In consideration of the benefits, a number of participants expressed that it would have been beneficial if they had access to it earlier in their LC journey. These findings are consistent with findings of previous OHP trials delivered to people with other chronic medical conditions.^36^, ^58^, ^59^, ^60^ Further details on participants' views on the LC‐OHP are reported separately.^39^

### 3.1 Implications for practice

Research into LC is limited, thus identifying the experiences of people with LC^61^ is essential to inform and improve practice. Based on our findings and consistent with the associated literature^62^ we propose the following recommendations to improve support for those with LC:

1. Increase awareness and understanding of LC for HCPs by providing them with training and education, for example, using the LC‐OHP.
2. Train HCPs to use effective communication skills with LC patients.
3. Increase awareness of those affected by LC, that is, family and friends to improve support and reduce stereotyping and disbelief.
4. Provide avenues for early LC intervention, for example, early referral to comprehensive support programmes.
5. Increase sharing of information across HCPs.
6. Arrange appointments with the same GP where possible.
7. Enable patient‐driven plans to facilitate a smooth return to work.

### 3.2 Strengths and limitations

The strengths of this study include a cohort that spanned a wide range of ages, and social and professional backgrounds, and this study was governed by a data management committee that included two members of the public with LC experience. Rigour was further demonstrated in programme activities, including researcher training on programme delivery of the intervention, provision of ongoing supervision and debriefings, completion of a fidelity checklist, having discussions and agreements on final themes, and including a sample of participants' quotes. The limitation was that it affected the generalisability of findings as the majority of participants were British and of white ethnicity, thus the results may not reflect the illness experience of other ethnic groups, and also most of the participants were females. Another potential limitation was social desirability bias as participants interacted with the same researcher over a 3‐month period. However, it could be noted that this assisted with maintaining the consistency of programme delivery.

## 4 CONCLUSION

Our findings detail the lived experiences of people with LC. Participants described several symptoms and reported their struggle with daily activities, their interactions with HCPs and the wider community and various difficulties associated with managing their illness. Increasing awareness about LC for HCPs, family and friends was one of many recommendations made by participants. The LC‐OHP was generally seen as a good facilitator for gaining acceptance of current health conditions, for gaining more understanding of what participants can and cannot do in its control and for monitoring and planning daily activities. It was suggested that referral to comprehensive support programmes such as the LC‐OHP should be made during the early stages of the illness. Further recommendations to improve LC support are presented.

## AUTHOR CONTRIBUTIONS

**Hiyam Al‐Jabr**: Conceptualising; methodology; validation; formal analysis; investigation; resources; data curation; writing—original draft; writing—review and editing; visualisation; supervision; project administration. **David R. Thompson**: Conceptualising; methodology; validation; resources; writing—review and editing; supervision. **David J. Castle**: Conceptualising; methodology; validation; resources; writing—review and editing; supervision. **Chantal F. Ski**: Conceptualising; methodology; validation; formal analysis; investigation; resources; data curation; writing—review and editing; supervision; project administration; funding acquisition.

## CONFLICT OF INTEREST STATEMENT

The authors declare no conflict of interest.

## ETHICS STATEMENT

The study received ethical approval from the University of Suffolk research ethics committee and the Health Research Authority ethical committee in November 2021. All participants provided written informed consent before starting the study.

## Data Availability

The data that support the findings of this study are available from the corresponding author upon reasonable request.
